# Long-lived photoexcitation probed by photo-induced enhanced Raman spectroscopy: unveiling charge dynamics in Ag–TiO_2_ nano-heterojunctions

**DOI:** 10.1038/s41598-025-89110-0

**Published:** 2025-02-15

**Authors:** Łukasz Pięta, Aneta Kisielewska, Adrian Warzybok, Ireneusz Piwoński, Kamilla Malek

**Affiliations:** 1 Faculty of Chemistry, Department of Chemical Physics, Jagiellonian University in Krakow, Gronostajowa 2, 30-387 Cracow, Poland; 2 Doctoral School of Exact and Natural Sciences, Jagiellonian University, Prof. St. Łojasiewicza 11, 30-348 Cracow, Poland; 3 Faculty of Chemistry, Department of Materials Technology and Chemistry, University of Lodz, Pomorska 163, 90-236 Lodz, Poland

**Keywords:** Photo-induced enhanced Raman spectroscopy (PIERS), Nanomaterials, Plasmonics, Silver nanoparticles, Thin titania film, Chemical engineering, Nanoscale materials

## Abstract

This work explores Photo-Induced Enhanced Raman Spectroscopy (PIERS) as a tool to investigate charge carrier dynamics in nanometer-thick Ag–TiO_2_ heterojunctions with a Schottky barrier. Due to the light-induced charge transfer process at the semiconductor–metal interface, PIERS provides a significant signal enhancement over traditional Surface-Enhanced Raman Spectroscopy (SERS). In turn, a remarkably stable PIERS signal lasting over 10 days after UVC light illumination cannot be explained exclusively by the presence and the lifetime of the induced oxygen vacancies, so other features of the Ag–TiO_2_ heterojunction must be responsible for this effect. Time-resolved Raman spectroscopy, photoluminescence (PL), UV–Vis, XPS, and I–V characterization were used to explore charge migration mechanisms further to prove PIERS applicability. While PL showed rapid healing of oxygen vacancies, the correlation of the PIERS signal with changes in the Schottky barrier height and relative changes in the electron density under various lighting conditions indicates that both Hot Electron Injection (HEI) and Plasmon-Induced Resonance Energy Transfer (PIRET) are responsible for the Raman signal changes over time. We showed that both phenomena can be probed by in situ PIERS spectroscopy. This proof-of-principle paves the way for developing more advanced photoactive semiconductor–metal composites suitable for energy conversion or storage, as well as SERS and PIERS analytics.

## Introduction

Coupled semiconductor–noble metal systems are being examined in various fields, such as photocatalysis, conversion of solar energy, photovoltaic devices, photoelectrocatalysis, and water splitting^1–4^. Enhanced photocatalytic efficacy of this kind of photocatalyst results mainly from two phenomena, i.e. (1) the formation of the Schottky barrier (φ) that prolongs the lifetime of photo-induced electrons, and (2) the generation of surface plasmon resonance and photonic enhancement via plasmon interaction with the semiconductor in the near-field^5,6^. In addition, these composite nanomaterials serve as powerful substrates for enhancing molecular detection in terms of Surface-Enhanced Raman scattering (SERS) where the electromagnetic field is enormously amplified by the synergic contribution of surface plasmons within metal nanoparticles (electromagnetic mechanism, EM), and the charge-transfer events between the adsorbed molecule and the substrate (chemical effect, CE). These nanomaterials are also useful for analytical SERS applications because photocatalytic properties of the semiconductor (SC) can be used for analyte degradation, making the SERS substrate reusable^7,8^. The additional UV light pre-treatment of the metal–semiconductor nanomaterial gives rise to an effect amplifying SERS signal and is called Photo-Induced Enhanced Raman Spectroscopy (PIERS)^9^. Most of the PIERS studies have focused on the proof-of-principle of this phenomenon across various semiconducting materials and Ag/Au nanoparticles (NPs) and their applicability as a sensing technique^9^. So far, the key mechanism responsible for this enhancement is related to the presence of defects, particularly oxygen vacancies (V_O_), designed in the semiconductor by vacuum annealing, inert-gas etching, or formed due to UV-light irradiation^10^. The generation of these atomic-scale defects facilitates charge transfer (CT) processes from the SC to the metal and the molecule upon illumination of the system with the visible laser light (a laser in the Raman spectrometer). Therefore, the enhancement factor (EF) of PIERS and its decay are the parameters for in situ monitoring of the V_O_ presence and lifetime in photocatalytically active materials^10,11^. In addition, a high PIERS enhancement factor can be achieved by an increase in the intrinsic polarizability of the resonant Raman probes upon charge transfer^12^. In most investigated SCs and their composites, the PIERS signal was observed for up to one hour and correlated with V_O_ healing. However, a work by Brognara and co-workers^13^ on AuNPs embedded in a tree-like TiO_2_ nanostructure reported an 8-day PIERS signal after UVC light pre-irradiation. An analysis of cathodoluminescence and LSPR indicated the formation of a narrow depletion zone at the SC-metal interface, and back-transfer charges onto AuNPs sustained the Raman signal. A key element of this long PIERS effect was the growth of the metal nanoparticles onto the SC since it was not observed for the NPs drop-casted method. We also observed a long PIERS signal (4 h) in our previous work on a thin TiO_2_ film on which AgNPs were grown under controlled photoreduction conditions to investigate the effect of their morphology and the plasmonic properties (LSPR, localized surface plasmon resonance)^14^. An impact of pre-irradiation UV wavelengths was also examined. We concluded that the PIERS amplification increases when the difference between the SC absorption and AgNP LSPR bands changes from ca. 80 to 40 nm and, the number of the metal nanoparticles exceeded 15% of the SC coverage.

Therefore, we designed herein a new composite characterized by the overlap between LSPR and the SC absorption band. We also conducted experiments to explore other possible mechanisms in photoactive materials, observable via in situ Raman spectroscopy. We determined the impact of light irradiation from the ultraviolet and visible regions of the solar light on the direct TiO_2_–Ag contact achievable by the photoreduction of Ag^+^ on the surface of the photoactive SC. This nanomaterial with a Schottky barrier and depletion zone at the SC—metal interface has already shown photocatalytic properties through the decomposition of organic molecules and the degradation of bacteria^15,16^. Photo-induced enhanced Raman spectroscopy was the primary tool for observing the efficacy of charge generation and its transfer between the components of the nanostructured material induced by UVC light illumination. The observed long PIERS cannot be explained only by the lifetime of the oxygen vacancies, detected here using photoluminescence spectroscopy. We also explored the effect of the Schottky barrier as an effective trap for photo-generated electrons and LPSR shifts caused by UV, Vis, and solar light illumination, which served as indicator of charge transfer. We proved the presence of additional mechanisms observed in PIERS, i.e., plasmon-induced resonance energy transfer (PIRET) and hot electron injection (HEI) that improve photoconversion in the SC-metal heterojunction.

## Experimental

### Materials

Titanium tetraisopropoxide (TTIP, 97%) and 4-mercaptobenzoic acid (4-MBA) were purchased from Sigma-Aldrich. Hydrochloric acid (HCl) was sourced from Chempur (Poland). Isopropanol (i-C_3_H_8_O, 99.7%), silver nitrate (AgNO_3_, 99.85%), and ethanol (C_2_H_5_OH, 99.8%) were acquired from Avantor Performance Materials Poland S.A. All chemicals were used as received without further purification. The water used in the experiments was purified using a Millipore simplicity UV system, possessing a resistivity of 18.2 MΩ/cm at 25 °C. Single-sided polished silicon wafers Si (100) were procured from the Institute of Electronic Materials Technology (ITME, Warsaw, Poland).

### Preparation of Ag–TiO_2_ composite nanomaterial

TiO_2_ thin films were fabricated on silicon wafers using the sol–gel and dip-coating methods outlined in the reference^17^. In brief, TTIP (1.25 ml) was introduced into i-C_3_H_8_O (16.6 ml) with vigorous magnetic stirring for 5 min. Subsequently, 40 µl of 2 M HCl, serving as a catalyst, was gradually incorporated. The resulting mixture was continuously stirred at room-temperature for an additional 30 min. Ultimately, the sol was applied to a silicon wafer utilizing a dip-coating technique, maintaining a constant immersion and withdrawal rate of 25 mm/min. The as-prepared TiO_2_ films were dried at 100 °C for 2 h, followed by calcination at 500 °C for 2 h. Next, silver nanoparticles were photochemically synthesized on the TiO_2_ film upon reduction of silver ions under UV illumination. The TiO_2_ coatings were placed in polymethacrylate cuvettes (1 × 1 × 4.5 cm), immersed in a 2.5 ml aqueous 1 mM solution of AgNO_3_ and exposed to UV light for 15 min. (UV-Consulting Peschl, 2 × 15W, λ = 365 nm, power density: 5 mW/cm^2^).

### Characterization of the Ag–TiO_2_ substrate

The morphology of the Ag–TiO_2_ substrate was characterized using a field emission scanning electron microscope (Hitachi S-4700). The average size of AgNPs (> 1000 NPs) and their surface coverage were calculated using ImageJ software (Wayne Rasband and contributors, National Institutes of Health, USA, Version 1.53 k). UV–Vis reflectance spectra were collected using a Lambda 35 UV/Vis spectrophotometer equipped with a Labsphere RSA-PE-20 Reflectance Spectroscopy Accessory (Perkin Elmer, Inc., USA). Spectra were acquired in the 200–800 nm spectral range with a scan speed of 240 nm/min. Reflectance conversion to the Kubelka–Munk function was performed using PerkinElmer UV WinLab software. All spectra were smoothed using a Savitzky-Golay protocol (13 points) and maxima of LSPR bands were determined using OPUS software (Bruker Optics, Bullerica, MA, USA, Version 7.2.139.1294).

The X-ray photoelectron spectra (XPS) were obtained using a PREVAC photoelectron spectrometer with a hemispherical VG SCIENTA R3000 analyzer. A monochromatized aluminium (Al) source (E = 1486.6 eV) was employed (with an angle of incidence of 20°) for the photoelectron spectra measurements, along with a low energy electron flood gun (FS40A-PS) to neutralize the surface charge of the samples. The analysis chamber maintained a base pressure of 5 × 10⁻^9^ mbar during measurements. Spectra of non-irradiated and irradiated (UV 254 nm, 45 min) Ag–TiO_2_ substrates were captured with a constant pass energy of 100 eV, both for survey scans and high-resolution spectra. The binding energies were calibrated using the C 1 s peak at 284.8 eV. High-resolution spectra fitting was performed using CasaXPS software.

The current–voltage (I–V) curves of the Ag–TiO_2_ sample were measured using a Mini Photoelectric Spectrometer equipped with a 4.30 controller operating in the potential range from − 2 V to + 2 V and a current resolution of 1 pA (Instytut Fotonowy, Krakow, Poland). Measurements were performed in dark conditions and after UV illumination (external UV lamp, λ = 254 nm, 5 W/m^2^) for 45 min. Relaxation measurements were performed every hour up to 4 h after turning off the ultraviolet light. All measurements were performed at ambient conditions.

Solid-state photoluminescence of Ag–TiO_2_ substrates was monitored using an FS5 UV–Vis-NIR spectrofluorometer (Edinburgh Instruments) equipped with a xenon arc lamp (150 W) as an excitation source (λ_exc_ = 300 nm) and an R928P Hamamatsu photomultiplier as a detector. Room-temperature spectra were collected using a standard holder dedicated to solid-state samples. Background corrections were applied using Fluoracle software procedures (Edinburgh Instruments). Time-resolved measurements were carried out within 1 h with a time step of ca. 1 min (in total 50 spectra).

### Raman microscopy

A 1 µM methanolic solution of 4-mercaptobenzoic acid (4-MBA, Sigma-Aldrich) was used as the probe molecule for Raman scattering. 40 µL of this solution was deposited on the substrate and allowed to dry on a hot plate (40 °C) for 4 min before collecting SERS spectra and after UV pre-illumination in the PIERS experiment. For the latter, the Ag–TiO_2_ substrates were exposed to UV radiation at 254 nm for 45 min, with the distance between the UV lamp and the substrate adjusted to achieve a power of 5 W/m^2^. No Raman signal was recorded when a 1 µM 4-MBA solution was deposited on a neat TiO_2_ film, neither before nor after UV irradiation.

Raman data was acquired using a confocal Raman microscope (WITec Alpha 300R Raman microscope, WITec, Germany) with a charge-coupled device (CCD) detector and a 600-grooves/mm grating. A solid-state laser with an excitation wavelength of 532 nm, coupled to the microscope by an optical fiber with a diameter of 50 µm, was used with a minimal laser power of 1.5 mW to prevent thermal degradation. Samples were illuminated using an air objective with a magnification of 40 × (NA: 0.6) and an exposure time of 1 s. Spectra were acquired in the 50–4000 cm^−1^ range with a spectral resolution of ca. 3 cm^−1^. At least six Raman images were recorded per sample from a 40 µm × 40 µm area with a 5 µm step size in the x–y plane, probing an area of approximately 10,000 µm^2^ per sample. Spectra were pre-processed by routine cosmic ray removal and background subtraction using WITec Project 5.1 software (WITec, Germany). SERS and PIERS spectra were collected under the same instrumental parameters. Raman images were preprocessed with Hierarchical Cluster Analysis (HCA) to reduce hyperspectral data sets and extract 30 mean spectra per image (CytoSpec v1.4.02). To assess the enhancement factor of SERS and PIERS^14^, the integral intensity of the 1589 cm^−1^ band was computed using OPUS software, and mean values were used for further data analysis.

## Results and discussion

### Structure and electronic states of the Ag–TiO_2_ composite material

**Fig. 1:**
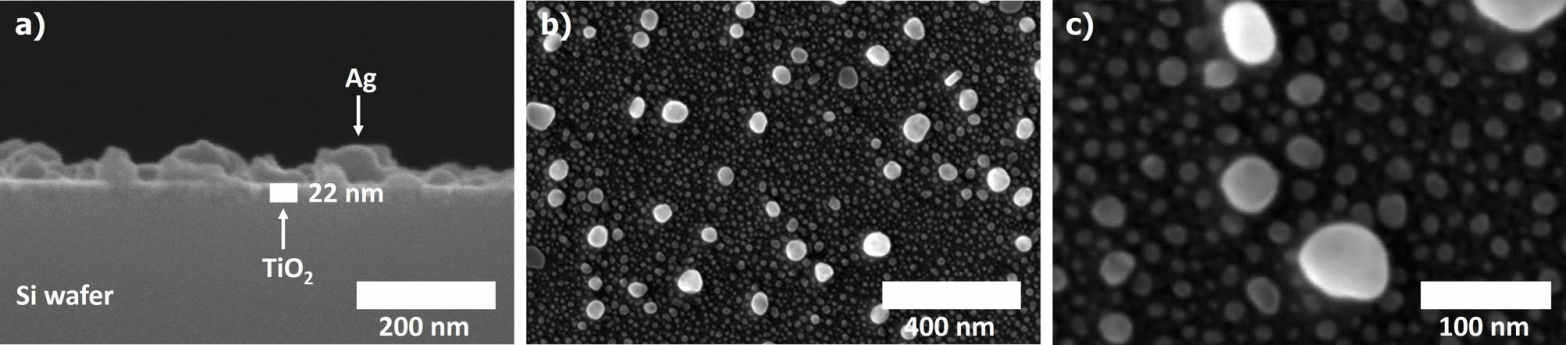
SEM images of a cross-section (**a**) and the surface of the Ag–TiO_2_ substrate (**b**, **c**) with a magnification of 100 000, 50 000 and 100 000, respectively.

**Fig. 2:**
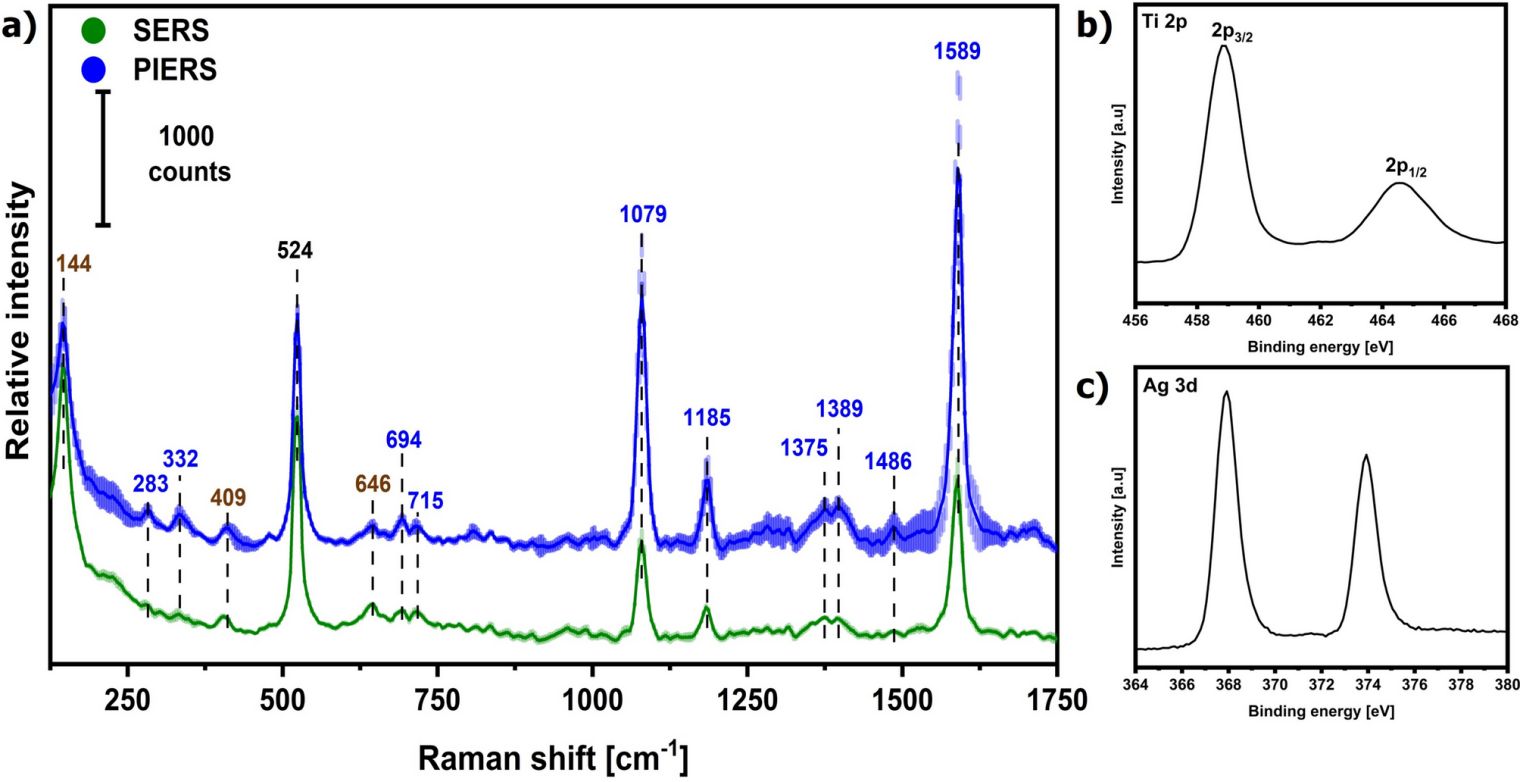
(**a**) Averaged (n = 380) SERS and PIERS spectra of 1 µM 4-MBA deposited on the Ag–TiO_2_ substrate. Bands labeled in brown and black originate from anatase and Si wafer, respectively. Shading denotes ± SD (standard deviation). High-resolution XPS spectra of (**b**) Ti and (**c**) Ag elements in Ag–TiO_2_ substrate.

The employed conditions in the sol–gel dip-coating method of the Ag–TiO_2_ fabrication yielded an anatase-TiO_2_ film with a thickness of 22 nm (± 3 nm) (Fig. 1a). The presence of anatase-TiO_2_ was confirmed by Raman (Fig. 2a) and XPS spectroscopy (Fig. 2b)^18,19^. The high-resolution spectrum of the Ti 2p doublet shown in Fig. 2b exhibited two peaks at 458.85 and 464.55 eV attributed to Ti^4^⁺ 2p_3/2_ and Ti^4^⁺ 2p_1/2_, respectively. The binding energy difference of 5.7 eV for this doublet confirms the presence of the anatase phase in the SC.

The photoreduction of silver ions produced oval Ag nanoparticles with an average diameter of 14 ± 5 nm on the SC film (Fig. 1b,c). A small fraction of AgNPs with a diameter greater than 40 nm was also observed. The nanoparticles were evenly distributed and spaced across the TiO_2_ surface, indicating a high degree of homogeneity of the surface coverage (Fig. 1b). The average number of AgNPs per µm^2^ was approximately 1500 (N_NPs_/μm^2^), giving 34% coverage of the TiO_2_ film. Figure 2c presents the XPS spectrum of Ag 3d. The peaks at 367.92 eV (3d_5/2_) and 373.92 eV (3d_3/2_), with a binding energy difference of 6.0 eV, confirmed that the nanoparticles are in the metallic state, Ag⁰ and not oxidized.

**Fig. 3:**
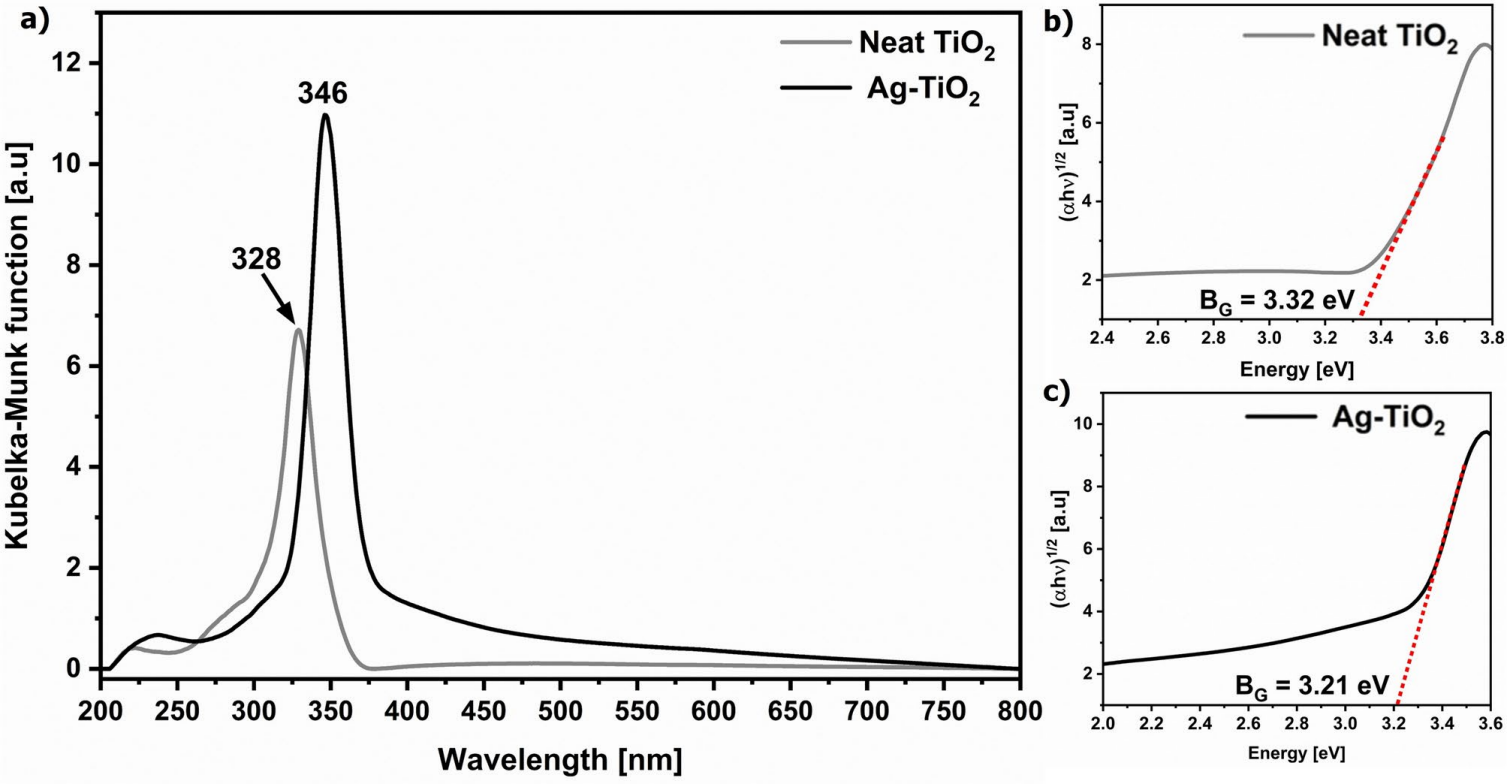
Diffuse reflectance spectra of the neat TiO_2_ film (**a**) and the Ag–TiO_2_ substrate (**b**). The insets show Tauc plots used to estimate the energy gap. The localized surface plasmon resonance of AgNPs in the 350–450 nm region partially overlaps with the absorption band of TiO_2_.

One of the most characteristic parameters of the semiconductor is the band gap. For this purpose, a maximum at 328 nm in the diffuse reflectance spectrum of the neat anatase TiO_2_ film was used to estimate band gap energy following a Tauc equation^20^ which was 3.32 eV (Fig. 3b). The deposition of AgNPs resulted in an 18 nm red-shift of this band that overlapped with localized surface plasmon resonance as indicated by the increased background in the 350–450 nm region (Fig. 3a). As a result, the energy gap of the Ag–TiO_2_ substrate decreased to 3.21 eV (Fig. 3c), possibly enhancing light absorption at the edge of the Vis region. The configuration of energy levels in the studied Ag–TiO_2_ nanomaterial is influenced by the emergence of interface or surface states within the band gap, attributed explicitly to forming Ag–O bonds as reported earlier^21^. Consequently, the effective energy of the band gap is diminished, as in the case studied here. Furthermore, the direct photoreduction of Ag^+^ ions on the TiO_2_ fostered the formation of Ag–O bonds, which influenced the metal–semiconductor interface and shaped the observed band structure.

**Fig. 4:**
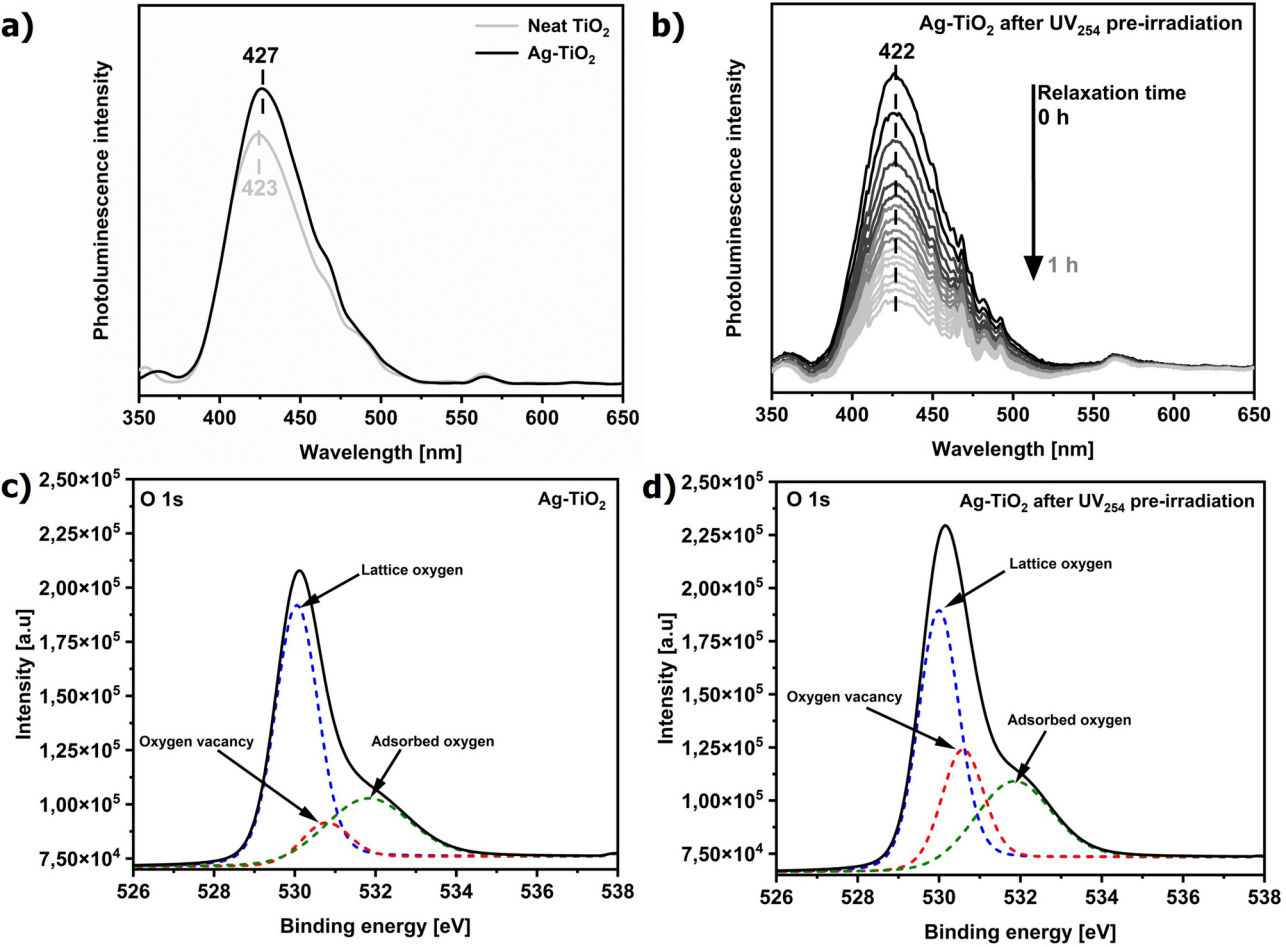
Photoluminescence spectra of (**a**) the neat TiO_2_ film and Ag–TiO_2_ substrate and (**b**) time-resolved Ag–TiO_2_ spectra after its 254 nm irradiation for 45 min. High-resolution XPS spectra of O element in Ag–TiO_2_ substrate (**c**) prior and (**d**) after UV irradiation.

To further investigate the electronic properties of the prepared Ag–TiO_2_ composite, photoluminescence (PL) spectra were analyzed (Fig. 4a,b). Both PL spectra of TiO_2_ and Ag–TiO_2_ exhibited a similar pattern but differed in the intensity of a broad band at 425 nm. This band was assigned to defect states (DS), including oxygen vacancies (V_O_), present directly below the conduction band (CB)^22–24^. High-resolution XPS O 1 s spectra confirmed the presence of oxygen vacancies in the substrates (Fig. 4c)^25^. Figure 4d shows that the peak intensity of V_O_ increases for Ag–TiO_2_ exposed to UVC light for 45 min. This indicated the generation of additional oxygen vacancies in the semiconductor structure during UV pre-irradiation. No significant changes were observed in the XPS spectra of Ag 3d and Ti 2p after UV substrate irradiation (data not shown). The decoration of the semiconductor film by the metal NPs and the formed Ag–O bonds contributed to higher photoluminescence by generating additional defect and interface states (IFS) at the Ag–TiO_2_ interface. A greater number of energy states below the conduction band of TiO_2_ increased the rate of recombination transitions, enhancing photoluminescence efficiency (Fig. 4a).

**Fig. 5:**
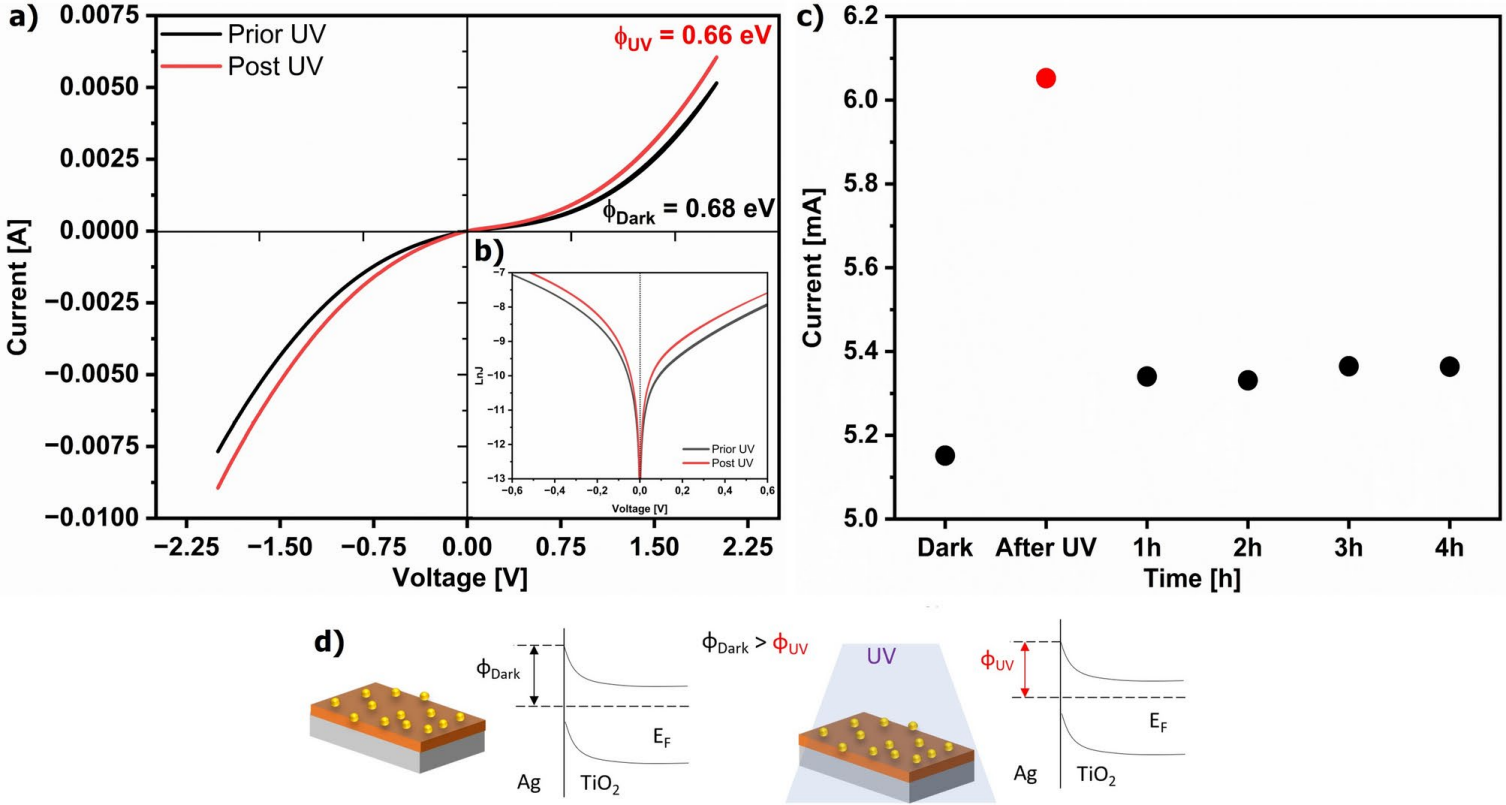
(**a**) I–V and (**b**) lnJ–V characteristics of the Ag–TiO_2_ heterojunction before (black line) and after (red line) UV pre-irradiation (254 nm, 5 W/m^2^, in air); (**c**) Current changes of the Ag–TiO_2_ heterojunction at a potential of 2 V, prior to and after UV pre-irradiation. (**d**) A schematic of slightly lowering the Schottky barrier for the Ag–TiO_2_ heterojunction after UV irradiation at ambient conditions.

The I–V characteristics for the Ag–TiO_2_ substrate established at ambient conditions were used to determine the Schottky barrier height according to the thermionic-emission-based diode equations (Fig. 5a):

1 $$J\left(T,V\right)={J}_{s}\left(T\right)[\mathit{exp}\left({\text{eV}}/{K}_{B}T\right)-1]$$

2 $${J}_{s}\left(T\right)={A}^{*}{T}^{2}\mathit{exp}\left(-e\varphi /{K}_{B}T\right)$$

were J(T,V) is the current density across the Ag–TiO_2_ interface, V—applied voltage, K_B_—Boltzmann’s constant, T– absolute temperature, J_s_(T)—saturation current density, A^*^—the Richardson constant equal to 108 A cm^−2^ K^−2^ for TiO_2_, e—a unit of elementary charge and φ—zero-bias barrier height. To estimate the barrier height of Ag–TiO_2_ before and after UV illumination, the lnJ-V curves were plotted according to Eq. 1 (Fig. 5b), and J_s_ was determined from Eq. 2^26^. The calculated barrier height (φ_dark_) of 0.68 eV aligned with those reported by Gao et al. and indicated the presence of the depletion zone at the TiO_2_-Ag interface^27^.

### Photo-induced charge carrier dynamics

The Raman spectrum of 1 µM solution of 4-MBA recorded after 45 min of photo-induction at 254 nm (PIERS) showed a considerable increase in the intensity compared to the SERS spectrum (Fig. 2). The two most intense bands at 1589 and 1079 cm^−1^ were attributed to the ring stretching (ν_8a_) and breathing (ν_12_) vibrations, respectively^28^. The CH deformation modes appeared at 342 and 1185 cm^−1^, while the deprotonation of the carboxylic group was indicated by bands in the 1360–1400 cm^−1^ region. The absence of bands at 277, 635, and 2570 cm^−1^ corresponding to -SH groups, and the presence of the 283 cm^−1^ band [ν(Ag–S)] confirmed the chemisorption of 4-MBA and the formation of self-assemble monolayer onto the entire substrate^14^. The elevated intensity of the ν_8a_ and ν_12_ modes also indicated a parallel orientation of the phenyl ring to the surface. No band shifts or the appearance of new bands of 4-MBA upon the illumination with either Vis or UV light confirmed its chemical stability. The enhancement factors (EF) calculated for the ν_8a_ mode were 1.8 × 10^4^ and 8.4 × 10^4^ for SERS and PIERS, respectively, and showed ca. 5-time amplification of the Raman signal due to the action of the UV light. The EFs were calculated for ca. 2.3 × 10^5^ molecules of the Raman probe in a laser spot. The EF calculated from the intensities of other 4-MBA bands did not differ considerably, so we excluded the changes in intrinsic Raman polarizability as a contributing factor to the PIERS effect, as observed in^12^.

**Fig. 6:**
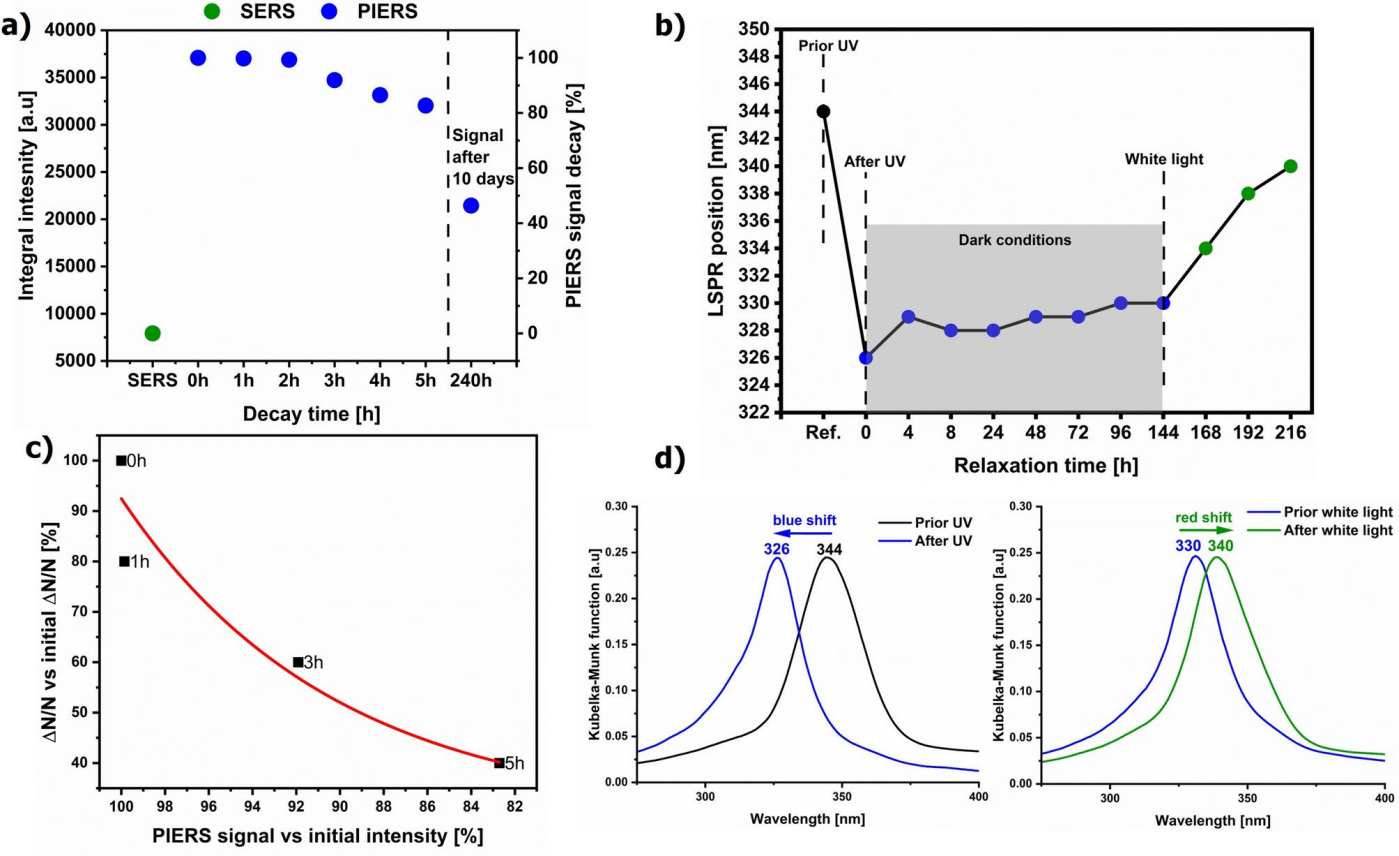
(**a**) The decay of the PIERS signal within 10 days after the 45 min UV pre-irradiation. (**b**) Shifts of the LSPR position due to storage of the Ag–TiO_2_ substrate under different light conditions. (**c**) Relationship between electron density changes on AgNPs and PIERS enhancement within 5 h after the UV pre-irradiation. (**d**) Diffuse reflectance spectra for specific measurements. The colors of the spectra correspond to those in (**b**).

In SERS, the plasmonic electromagnetic effect, as well as Ag-to-4-MBA charge transfer, contributed to surface enhancement^29^. In PIERS, additional enhancement resulted from direct excitation of the semiconductor with UV light and the generation of oxygen vacancies. Due to Vis excitation, electrons migrate from the defect states, including V_O_, to CB, increasing the charge transfer rate to the metal and adsorbed molecules^7,11,28–33^. However, our observation of the PIERS decay process indicated that other processes, beyond V_O_ generation, must contribute to the PIERS enhancement. The PIERS signal was probed in 1-h periods up to 5 h and 10 days after switching off the UV light (Fig. 6a). It was remarkably stable over 5 h, decreasing only by ca. 20%. After 10 days, it dropped by 50% compared to t = 0 h and did not reach the SERS level.

To assess the role of the V_O_ states, we first examined their PL band at 425 nm within one hour after the UV pre-illumination of the substrate since this relaxation time at the ambient conditions has been proposed so far (Fig. 4b)^11,34^. The UV pre-illumination generates the additional oxygen vacancies in the TiO_2_ structure (Fig. 4d), and Vis illumination from the Raman laser excites electrons from the defect states to the conduction band and further to AgNPs and the molecules. A gradual decrease in the PL intensity within 1 h showed the progressive disappearance of V_O_ under oxygen conditions in contrast to the almost constant PIERS signal in that period (Figs. 4b and 6a). This indicated that the V_O_ healing process cannot be exclusively correlated with the 10-day persistence of the substantial photo-induced Raman enhancement on the designed Ag–TiO_2_ junction. Furthermore, the non-zero PL intensity after 1 h still exhibited the defect and interface states that still promoted the transfer of electrons from these levels into the conduction band of TiO_2_ and further to AgNPs due to the laser Vis excitation in the PIERS experiment. Subsequently, the migration of the excited electrons effectively extended the high PIERS enhancement over time.

Next, the Schottky barrier at the Ag–TiO_2_ interface preserved the outflow of the hot electrons from the metal to the semiconductor (Fig. 5a,b). After the UV light pre-illumination (φ_UV_), its height slightly decreased to 0.66 eV at ambient conditions (Fig. 5a). After 4-h relaxation, the I–V characteristic of the Ag–TiO_2_ heterojunction was coming towards values without pre-illumination, however, it did not return to its initial position (Fig. 5c). Changing the I vs V characteristic of the Ag–TiO_2_ heterojunction led to a current reduction compared to the current value before UV exposure (Fig. 5c). Indeed, the stability of the Schottky barrier after UV light action and in the relaxation time supported the accumulation of electrons at AgNPs. These results implicated the permanence and stability of the tested system, in that the Schottky barrier effectively prevented electrons from flowing out of the metal, regardless of lighting conditions and time.

The third key process required to maintain long-lasting PIERS is Plasmon-Induced Resonance Energy Transfer (PIRET). In this scenario, the non-radiative decay of LSPR on the metal induces the local excitation of the semiconductor, leading to the generation of electron–hole pairs and again the subsequent transfer of electrons from VB to CB, and then to AgNPs, amplifying the PIERS effect. According to studies on the photocatalytic efficacy of plasmonic nanoparticle-semiconductor composites, the PIRET effect is substantial when the LSPR of the metal NPs overlaps the absorption band of the SC and a tight metal-SC heterojunction is present, like in our case (Fig. 3a)^2,5,6,35^. For this reason, the PIERS signal in the fifth hour decreased only by ca. 20% (Fig. 6a).

We also investigated the electronic properties of the Ag–TiO_2_ nanomaterial by diffuse reflectance spectroscopy after the UV pre-treatment and in different light conditions to probe the charge accumulation efficiency in AgNPs (Fig. 6b,d). Briefly, the Ag–TiO_2_ substrate was first photo-activated with the 254 nm UV light for 45 min, as in the PIERS experiment (before and after UV data in Fig. 6b,d). Then, the sample was stored in the dark for 144 h (6 days), and the spectra were collected in some time intervals (dark conditions in Fig. 6b). After that, the substrate was exposed to the white light, and the spectra were measured daily for the next three days (white light data in Fig. 6b,d). The UVC pre-illumination caused charge transfer from the TiO_2_ film to AgNPs shown by an 18 nm blue-shift of the LSPR maximum (Fig. 6d). This process amplified the electromagnetic field, enhancing Raman scattering of the surface-adsorbed molecules. In addition, the accumulated electrons increased the CT rate between the silver and 4-MBA. The 6-day lack of light did not cause considerable changes in the LSPR position, indicating effective trapping of photo-induced charge due to the presence of a Schottky barrier at the Ag–TiO_2_ interface (Fig. 6d). Exposure to the white light induced the outflow of electrons from Ag to TiO_2_, as the LSPR maximum was red-shifted by 10 nm within 24 h gradually achieving the position observed for the non pre-illuminated substrate (Fig. 6b,d). We estimated relative changes in the electron density for the selected time intervals of our experiment according to the equation proposed by Mulvaney et al.^36^:

$$\frac{\Delta N}{N}=-\frac{2\Delta \lambda }{{\lambda }_{0}}$$

where ∆N is the injected electron density, ∆λ is the measured LSPR shift after UV irradiation, and λ_0_ is the initial position of the LSPR. The initial electron density change after the substrate pre-irradiation was 10.5%, resulting in ca. fivefold enhancement of the PIERS signal compared to SERS. Electron density changes and PIERS EF in the 5-h decay process were exponential (Fig. 6c). This observation implicated the fact that the long-lasting and sustainable increased electron density on the nanoparticles is a key parameter for achieving high PIERS enhancement factors and making the PIERS technique a powerful tool for probing composite nanomaterials.

**Fig. 7:**
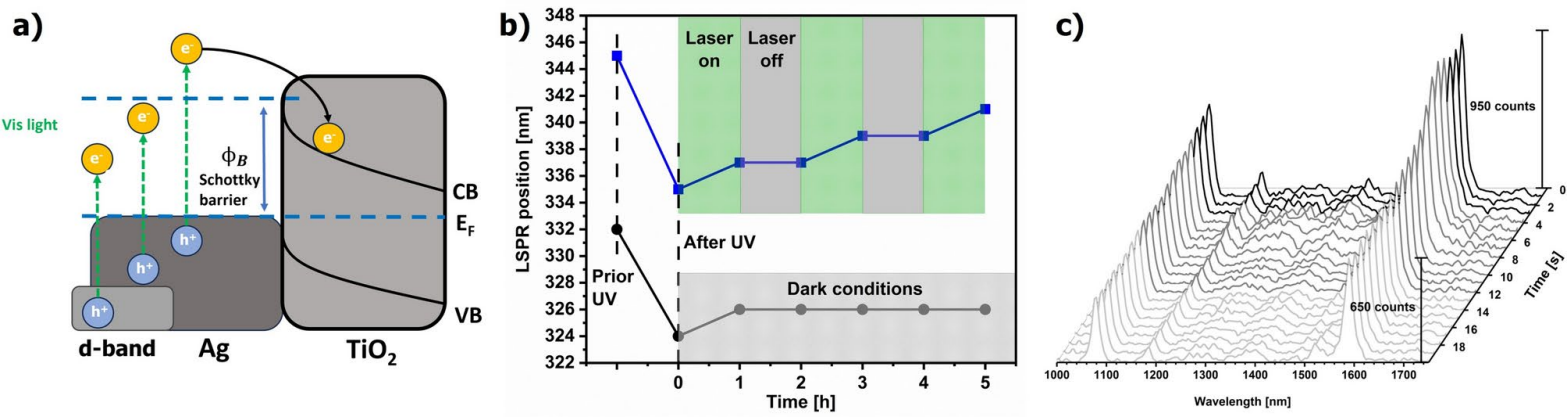
(**a**) A schematic of the HEI mechanism at the Ag–TiO_2_ interface. (**b**). Changes in the LSPR position of the Ag–TiO_2_ substrate upon the UV and laser light (532 nm) treatment. (**c**) A decay of the PIERS signal in the second scale due to the illumination by laser light (532 nm).

The most reasonable explanation for the observed decrease in electron density on AgNPs under visible light is related to the induction of a hot electron migration process (HEI) at the Ag–TiO_2_ interface (Fig. 7a).

Light absorption by AgNPs induces plasmons and excites electrons to higher energy states in the silver. These hot electrons can then tunnel through the Schottky barrier and transfer into the conduction band of TiO_2_. The hot electron injection phenomenon in metal–semiconductor composite nanomaterials has been extensively documented in the literature, particularly in photocatalysis and solar energy conversion^4,6,35–38^. This effect is the fundamental mechanism underpinning the functionality of plasmonic hot-electron photodetectors^25,39^. We simulated HEI by the exposure of the UV pre-illuminated Ag–TiO_2_ substrate to the laser light at 532 nm with a power of 1.5 mW used for the excitation of PIERS spectra and the effect was monitored through changes in the LSPR position in 1-h intervals (Fig. 7b). The entire substrate surface was continuously scanned with the laser spot for one hour and compared to the sample stored in dark condition. Again, the blue-shift of LSPR induced by the UV pre-illumination confirmed the charging of AgNPs. Regardless of the light conditions, both samples showed a slight red-shift of the band after one hour, which could be associated with the healing of oxygen vacancies observed by photoluminescence due to storing the substrates in the ambient atmosphere containing oxygen. After this time, the LSPR position remained constant in the dark. In contrast, a significant red-shift was observed after each cycle of the laser illumination (*laser on* data in Fig. 7b). Interestingly, no CT processes appeared after switching off the laser light (*laser off* data in Fig. 7b) indicating the immediate impact of the strong and focused visible light on the outflow of hot electrons from silver nanoparticles. In that way, many photons effectively induced plasmons in the metal nanoparticles, causing simultaneous excitation of electrons to higher energy states near the Fermi level. This, in turn, increased the rate of HEI and electron tunneling to TiO_2_ through the Schottky barrier in a relatively short time compared to HEI induced by white light (Fig. 6b).

Next, we tracked this effect by the collection of PIERS spectra from one spot within 1-s intervals for 20 s (Fig. 7c). Only the entire intensity of the spectra gradually decreased without alternations of band positions and relative intensities, confirming the lack of photodegradation of the Raman probe. After just 15 s of continuous laser illumination, the 4-MBA signal dropped by ca. 30%. The corresponding SERS experiment showed ca. 20% decrease in the Raman intensity after 20 s, implicating that HEI also contributes to the SERS response in metal–semiconductor nanomaterials, though its effect is more pronounced in PIERS. The greater intensity reduction over time for PIERS compared to SERS can be attributed to the UV pre-treatment that slightly increased the Fermi level in AgNPs. This increase allowed photo-generated hot electrons to more easily overcome the Schottky barrier.

## Conclusions

**Fig. 8:**
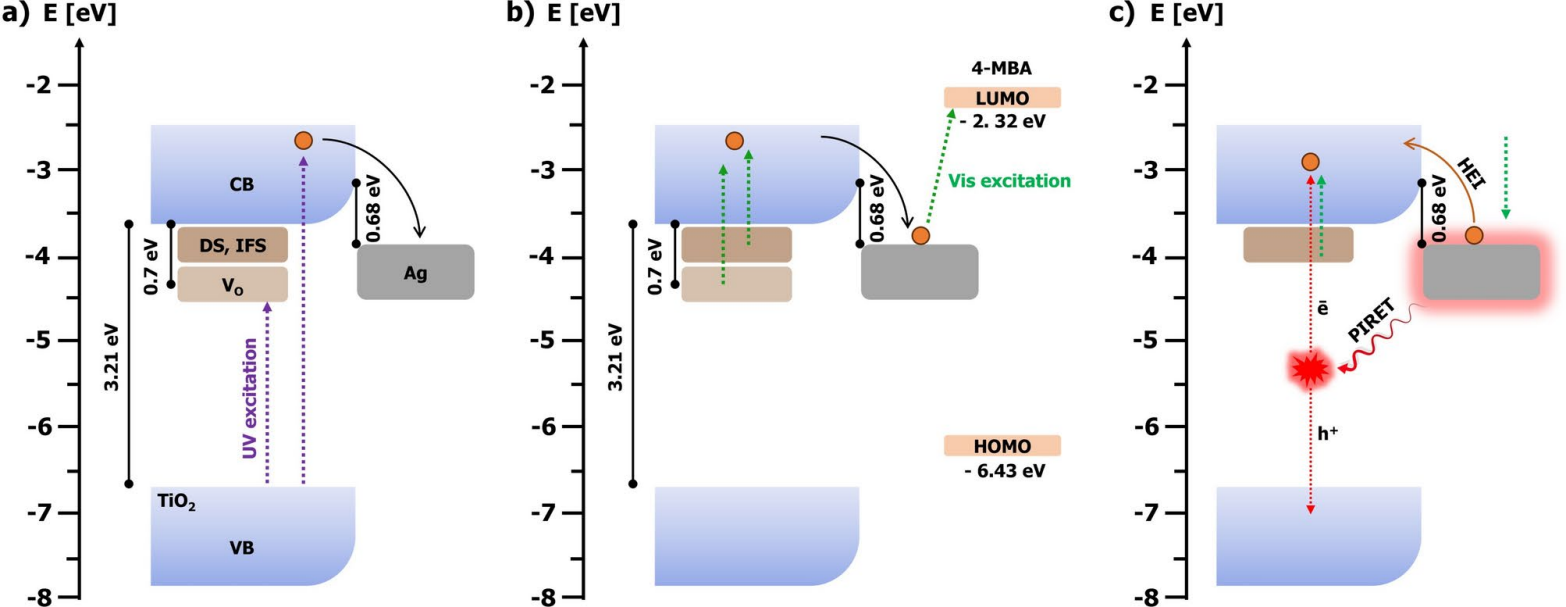
Proposed mechanism of PIERS and its decay in time for Ag–TiO_2_ heterojunction. (**a**) UV pre-irradiation: generates V_O_ and excites electrons in TiO_2_, which flow to the AgNPs through the Schottky barrier; (**b**) PIERS measurements: visible laser light causes electron transfer from defects, interface, and V_O_ states to the AgNPs. Accumulated electrons on AgNPs are excited to the LUMO level of the analyte; (**c**) After one hour of UV pre-irradiation, V_O_ are healed, but LSPR induced by visible light leads to the PIRET and HEI effects, which are responsible for the increasing and decreasing electron density on AgNPs, respectively.

This work unveils alternative pathways for charge transfer and accumulation within Ag–TiO_2_ nano-heterojunctions (Fig. 8). These pathways extend beyond the formation of surface oxygen vacancies in the semiconductor. By combining PIERS with different light sources, one can probe the dynamics and stability of these processes. The unique long-lived PIERS signal (10 days) arises from the interplay of several factors: (1) Schottky Barrier-Mediated Charge Storage—the presence of a Schottky barrier at the Ag–TiO_2_ interface hinders electron backflow from Ag to TiO_2_ (Fig. 8a), leading to a sustained increase in electron density within AgNPs, (2) Direct Photoreduction and Enhanced Charge Migration—photoreduction of silver ions on TiO_2_ facilitates the formation of a tight metal–semiconductor interface, promoting efficient charge migration and accumulation, as evidenced by the extended PIERS effect. Notably, other studies have reported over an hour of PIERS enhancement for composite materials prepared in this way^7,14,40^, (3) Light-Driven Excitation and Energy Transfer—the excitation of localized surface plasmons in AgNPs by visible light was responsible for the electromagnetic enhancement observed in both SERS and PIERS, however, the accompanying non-radiative decay of LSPR resulted in the generation of HEI and PIRET effects (Fig. 8c). Hot electron transfer from Ag to TiO_2_ reduces the electron density in AgNPs, contributing to lower charge transfer, and PIERS decay. Plasmon energy transfer from AgNPs to TiO_2_ excites the semiconductor, promoting electron transfer from its valence band (VB) to the conduction band. These electrons then migrate to AgNPs, further amplifying the PIERS effect, (4) Defect-Mediated Charge Transfer—defect states and interface states with energies similar to surface oxygen vacancies also prolong the PIERS duration (Fig. 8b). These states allow visible light excitation of electrons, facilitating their transfer to the TiO_2_ conduction band and subsequently to AgNPs. Importantly, all these mechanisms occur concurrently during PIERS measurements. The interplay of these factors determines the observed PIERS enhancement and its decay profile, providing valuable insights into the charge dynamics within the nano-heterojunction.

## Data Availability

The authors declare that the data supporting the findings of this study are available within the paper and Mendeley Data repository with the identifier Doi: 10.17632/2tgbfg825g.1. Should any raw data files be needed in another format, they are available from the corresponding author upon reasonable request.

## Abbreviations

4-MBA: 4-Mercaptobenzoic acid
B_g_: Band gap
CE: Chemical effect
CT: Charge transfer
CB: Conduction band
DS: Defect states
EF: Enhancement factor
EM: Electromagnetic mechanism
φ: Schottky barrier
HOMO: Highest occupied molecular orbital
IFS: Interface states
LUMO: Lowest unoccupied molecular orbital
LSPR: Localized surface plasmon resonance
NPs: Nanoparticles
PL: Photoluminescence
PIRET: Photo-induced resonant energy transfer
PIERS: Photo-induced enhanced Raman spectroscopy
SC: Semiconductor
SERS: Surface enhanced Raman spectroscopy
TTIP: Titanium tetraisopropoxide
VB: Valence band
V_O_: Oxygen vacancy
